# Serotonergic Genotypes, Neuroticism, and Financial Choices

**DOI:** 10.1371/journal.pone.0054632

**Published:** 2013-01-30

**Authors:** Camelia M. Kuhnen, Gregory R. Samanez-Larkin, Brian Knutson

**Affiliations:** 1 Finance Department, Kellogg School of Management, Northwestern University, Evanston, Illinois, United States of America; 2 Department of Psychology, Vanderbilt University, Nashville, Tennessee, United States of America; 3 Department of Psychology, Stanford University, Stanford, California, United States of America; University of Minnesota, United States of America

## Abstract

Life financial outcomes carry a significant heritable component, but the mechanisms by which genes influence financial choices remain unclear. Focusing on a polymorphism in the promoter region of the serotonin transporter gene (5-HTTLPR), we found that individuals possessing the short allele of this gene invested less in equities, were less engaged in actively making investment decisions, and had fewer credit lines. Short allele carriers also showed higher levels of the personality trait neuroticism, despite not differing from others with respect to cognitive skills, education, or wealth. Mediation analysis suggested that the presence of the 5-HTTLPR short allele decreased real life measures of financial risk taking through its influence on neuroticism. These findings show that 5-HTTLPR short allele carriers avoid risky and complex financial choices due to negative emotional reactions, and have implications for understanding and managing individual differences in financial choice.

## Introduction

Recent findings suggest that various aspects of economic behavior are heritable. Studies comparing choices of identical and fraternal twins find that inherited (and likely genetic) factors can account for 20%–30% of variation across individuals in terms of experimentally-elicited risk preferences [1], allocation to risky assets in real life portfolios [2], [3], and the propensity to save [4]. Further, genetic variation related to the functioning of two broadly distributed and influential neurotransmitters, serotonin and dopamine, have been shown to correlate with economic behavior in healthy individuals [5], [6], [7], and in individuals with diagnosed disorders including pathological gamblers and those with anxiety disorders [8], [9]. While these findings convincingly suggest that genetic factors are related to economic choice, they do not address the equally important question of *how* genes influence behavior. For example, do genes influence cognitive abilities, do they shape the way people learn in financial markets, or do they determine risk attitudes? We sought to address this question by focusing on the role of a genetic polymorphism in the promoter region of the serotonin transporter gene (5-HTTLPR) which has recently been identified as important for financial risk taking. Prior research suggests that the short and the long variants of this gene may have different effects on risk taking, and therefore on economic behavior. The short allele has been associated with higher scores on neuroticism and harm avoidance [10], [11], a stronger attentional bias towards negative stimuli [12], and lower life satisfaction [13], as well as with less risky experimentally elicited portfolio allocation choices [6]. Nonetheless, it is still unknown whether differences in financial choice documented in the laboratory generalize to real life choices and outcomes among community members, and if so, which mechanism underlies the risk avoidant choices of the short allele carriers. The goal of this study was to test whether short versus long serotonin transporter allele status would influence financial choices in a community sample, and to explore potential psychological mediators.

## Methods

The 60 subjects in this study (30 male, age range 20–85 years, mean age 54 years) were recruited by a survey research firm with the goal of being representative of the San Francisco Bay Area population. Data collection was conducted at Stanford University. Subjects gave written informed consent prior to participating. The study and consent procedure were approved by the Stanford IRB committee. For these individuals, we obtained demographic information, information regarding financial status (i.e., assets, debt and income), measures of cognitive ability and numeracy, measures of attitudes and beliefs concerning economic decisions and outcomes, and objective financial information from official credit reports. Subjects ability to learn from financial information was also measured using an investment task, described below. Summary statistics for these measures are presented in Table 1.

**Table 1:** Summary statistics.

	Mean	Std. Dev.	N
**Demographics, self-reported income, assets and debt**			
*Age*	54.13	18.11	60
*Male*	0.5	0.50	60
*Assets* (1–16 *scale)*	11.98	4.49	60
*Debt* (1–16 *scale)*	7.50	5.10	60
*Education* (*years*)	15.43	2.42	60
*Income* (1–12 *scale*)	6.67	2.56	60
**Cognitive abilities and learning in investment task**			
*Numeracy*	7.88	1.53	60
*Cognitive inflexibility* (*Trails B*–*A*)	35.73	15.10	60
*Working memory* (*letter* – *number sequencing*	10.35	2.84	60
%*Correct choices in gain condition*	0.62	0.31	49
%*Correct choices in loss condition*	0.66	0.20	49
**Financial choices**			
%*Allocation to stocks*	35.58	29.45	60
%*Allocation to bonds*	28.67	20.54	60
%*Allocation to cash*	35.75	30.52	60
*Involvment in finances*	2.30	0.77	60
**Beliefs**			
*Risk of stocks*	5.37	1.39	60
*Subjective status in US*	6.41	1.38	60
* Subjective status in local community*	6.27	1.60	60
**Credit report data**			
* Number of credit accounts*	19.03	11.69	31
* FICO score*	759.63	76.93	31
* Credit Ammount (mil$)*	0.24	0.34	31
* Amount Used (mil$)*	0.15	0.30	31
**Affect measures**			
* Neuroticism*	27.93	8.98	60
* Positive Affect*	3.00	0.52	60
* Negative Affect*	1.88	0.40	60

Sample summary statistics for subjects’ real life and experimental financial outcomes, demographic characteristics, and measures of cognitive and affect measures.

Salivary DNA was collected from all subjects (with a cheek swab), and genotyping of the 5-HTTLPR polymorphism was conducted according to standard protocols [10]. In this sample, 52% (32) subjects had the short/short (SS) genotype, 32% (19) had the short/long (SL) genotype, and 15% (9) had the long/long (LL) genotype. The distribution was consistent with that expected under Hardy Weinberg equilibrium (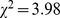, 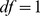, 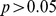). Within the sample, chi-square tests indicated that genotype did not significantly vary as a function of gender or ethnicity.

### Demographics and Life Financial Outcomes

A questionnaire was administered to assess the age, marital and occupational status, level of income, number of years of education, and ethnicity of the subjects, as well as their assets and debt. Household income was measured using a scale from 1 to 12, where 1 represented “less than $15,000” and 12 represented “higher than $500,000”. Assets were assessed with the question “What are your approximate current assets? (i.e., home value, bank accounts, investments, belongings)” using a 16-category ordinal response scale ranging from <$500.00 in the lowest category to >$1,500,000.00 in the highest. Debt was assessed with the question “What are your approximate current debts? (i.e., outstanding home loans, outstanding car loans, outstanding student loans, credit card debt, medical debt)” using a 16 category ordinal response scale ranging from <$500.00 in the lowest category to >$1,500,000.00 in the highest.

### Cognitive Ability and Numeracy

Subjects were also administered standard tests of cognitive ability. The Trail Making Test [14] assessed cognitive flexibility. The test has two parts (A & B) which are both timed until completion. The first part (Trails A) requires that subjects sequentially connect 25 encircled numbers (1, 2, 3, etc) that are randomly arranged on a sheet of paper. The second part (Trails B) requires that subjects connect a series of numbers and letters in an alternating pattern (1, A, 2, B, 3, C, etc.) that are randomly arranged on a sheet of paper. The score on this test is calculated as the difference between the time taken to complete Trails B versus Trails A, and indicates how easily individuals can alternate or switch between different activities. Higher scores correspond to less cognitive flexibility.

The Letter-Number Sequencing subtest from the Wechsler Adult Intelligence Scale [15] assessed memory capacity. An experimenter verbally listed a series of randomly ordered numbers and letters to the subject (e.g., C, 1, A, 6, 2) and asked the subject to repeat the series back in with the numbers listed first, in numerical order, followed by the letters in alphabetical order (e.g., 1, 2, 6, A, C). Performance on this test requires that subjects both maintain the series of randomly ordered numbers and letters in short term memory and manipulate the stored information by sorting the representation in memory before verbally repeating it. Researchers refer to this combination of short-term memory maintenance and manipulation as “working memory”. This measure of working memory correlates well with general intelligence.

A numeracy inventory assessed quantitative skills with basic number problems [16]. This 11-item measure contains questions such as: “The chance of getting a viral infection is.0005. Out of 10,000 people, about how many of them are expected to get infected?”. All of the questions are focused on computing probabilities or proportions. The measure is considered to index an individual’s ability to accurately compute numerical information about risk.

### Financial Choices

To assess subjects’ willingness to take financial risk, we asked: “If you suddenly received $10,000, how much would you allocate to each of the following? (out of 100%): a. equities: – % (includes stocks, mutual funds, or other equity components); b. bonds: – % (includes government bonds, municipal or corporate bonds, bond mutual funds, or other fixed-income components), c. cash: –% (includes money market accounts).” To measure subjects’ involvement in their finances, we asked the question: “How much experience do you have with investing?”. The possible answers were:(1) “I have had a savings account, but no other investments”; (2) “I have had investments other than a savings account (e.g., stocks, bonds, or mutual funds), but I do not tend to make my own decisions about those investments”; (3) “I actively make decisions about investing my money (e.g., in stocks, bonds, and other types of investments).”

### Beliefs

To index subjects’ beliefs about the risk of investing in equities, we asked: “Rate how risky you perceive this activity to be: Investing 5% of your annual income in a very risky stock”. The answer was an integer from 1 (“not at all risky”) to 7 (“extremely risky”).

### Socioeconomic Status

A subjective assessment of subjects’ socioeconomic status (SES) indexed their perceived standing in society and associated perceived control over life outcomes, since prior research suggests that individuals with low subjective SES perceive the world as less controllable [17]. Subjects saw a picture of a ladder along with instructions that read: “Think of this ladder as representing where people stand in the United States. At the top of the ladder are the people who are best off - those who have the most money, the most education and the most respected jobs. At the bottom are the people who are the worst off who have the least money, least education, and the least respected job or no job. The higher up you are on this ladder, the closer you are to the people at the top; the lower you are, the closer you are to the people at the bottom. Where would you place yourself on this ladder? Place an X on the rung where you think you stand at this time of your life relative to other people in the United States.” For local status, the instructions read: “Think of this ladder as showing where people stand in their communities. People define community in different ways. Please define it in whatever way is most meaningful to you. At the top of the ladder are the people who have the highest standing in their community. At the bottom are the people who have the lowest standing in their community. Where would you place yourself on this ladder? Place an X on the rung where you think you stand at this time of your life relative to other people in your community.” The answers can range from 1 to 10, where 1 represent the lowest rung on the ladder, while 10 represents the highest.

### Credit Report Data

We obtained credit reports for a subsample of 31 subjects, from which we extracted subjects’ overall FICO credit scores, and calculated the available credit amount and the percent of credit used as objective proxies of assets and debt, respectively. The correlations between these objective measures (using log values) and the subjective self-reported assets and debt were significant and robust (r = 0.56 for assets, and r = 0.85 for debt, 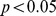), supporting the validity of the self-reported information.

### Financial Learning Task

This task indexed subjects’ ability to learn from financial gains or losses [18]. Gain and loss learning were separately assessed to account for the possibility that depending on subjects’ 5-HTTLPR genotype, they may differentially attend to, encode, or retrieve gain or loss information. Subjects made 24 choices between two risky assets in both gain and loss conditions. In each condition, the two assets were represented by a pair of abstract symbols. After choosing one of the assets from each pair, subjects saw the outcome associated with their choice. On average, one of the assets yielded a better outcome, while the other yielded a worse outcome. Specifically, in the gain condition the better asset had a 66% probability of yielding a $1 dividend and a 33% probability of yielding a $0 dividend. These probabilities were reversed for the worse asset (i.e., the $1 dividend had only a 33% chance of being obtained). In loss condition, the better asset had a 66% probability of yielding a $0 dividend and a 33% probability of yielding -$1.00, and outcome probabilities were reversed for the worse asset. Within each pair, assets appeared randomly and with equal frequency on the left or right side of the screen. Asset pairings with better or worse outcomes were randomly assigned by the computer at the start of the experiment and counterbalanced across subjects. Subjects were explicitly informed about the dividend distributions and instructed to try to maximize their earnings throughout the experiment by choosing the assets they believed to be the better ones. To quantify how well subjects learn from financial information, we calculated the fraction of trials in the gain condition and in the loss condition when subjects made the correct Bayesian choice, conditional on the information set available at the time. For this calculation, we excluded trials in which both options had equal chances of being optimal (including the first trial). For incentive compatibility, dividends accumulated during this task determined subjects’ payment.

### Affective Traits

Although a great deal of research has focused on the influence of transient emotional states (i.e., current levels of excitement or anxiety) on decision making [19], [20], relatively less research has focused on chronic emotional traits (i.e., how excited or anxious someone is on a regular basis). We measured trait differences in positive and negative emotions related to more stable measures of the affective style of an individual. This enduring style may have cumulative influences on choice behavior over the life course, since prior work suggests that experimentally-induced transient positive aroused affect can decrease perceptions or risk, whereas negative aroused affect can increase perceptions of risk [21], [22], suggesting that these transient “anticipatory” affective states can increase or decrease financial risk taking, respectively [23].

A 60-item short form of the Neuroticism, Extroversion/Introversion and Openness to Experience Personality Inventory (NEO-SF) [24] asked subjects to indicate their level of endorsement of each of 60 statements related to commonly-assessed personality traits. These common traits are Neuroticism, Extraversion, Openness to Information, Agreeableness, and Conscientiousness. Sample items for Neuroticism include: “I am (not) a worrier.” and “I often feel tense and jittery.” In this paper we focus on neuroticism for several reasons, including the strong link between this measure and anxiety, its high level of heritability (50% of individual differences in neuroticism are due to genetic factors), and its correlation with the 5-HTTLPR genotype [10], [11], [25]. High neuroticism scores indicate a high propensity to experience anxiety, worry, vulnerability and self-doubt.

We also administered the Affect Valuation Index [26] to assess the extent to which subjects experience and value positive and negative emotions on a regular basis. Subjects saw 30 emotional descriptors referring to either positive affect (e.g., enthusiastic, excited, happy) or negative affect (e.g., fearful, nervous, sad) and were asked to rate how often they actually experienced that feeling over the course of a typical week, using a scale from 1 (“never”) to 5 (“all the time”). For each subject, we calculated their average rating across words in the positive domain, and separately in the negative domain, and used these as proxies of the intensity of positive and negative affect that characterize that individual in general.

## Results

### Financial Choices

The regression models in Table 2 present the effects of having the SS or SL version of the gene, relative to having the LL version, on several financial choices and outcomes. In these regressions, we also included subjects’ age and sex as predictors of choices and outcomes. Subjects with the SS genotype choose to invest 23.84% less in stocks and choose to keep 25.60% more in cash than LL subjects (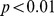). There was no significant difference between these groups regarding the percentage invested in risky bonds. Both SS and SL subjects reported that they were less involved in actively making financial decisions compared to LL subjects. Using the 1 to 3 scale of active financial involvement, SS subjects’ average involvement score was 0.45 points lower than that of LL subjects (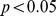). In the subsample of 31 subjects for whom credit reports were available, SS and SL subjects had approximately 13 fewer credit lines compared to LL subjects, and also had higher FICO scores. The difference in FICO score was 47 points higher for the SL subjects (not statistically significant) and 93 points higher for the SS subjects (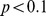).

**Table 2:** Genetic effects on financial choices and outcomes.

Dependentvariable	%StockAllocation	%CashAllocation	Involvementin Finances	Numberof creditlines	FICOscore
*fivehttlpr = = SL*	–9.18	3.12	–0.65	–13.43	47.16
	(–0.91)	(0.36)	(–3.16)***	(–2.10)**	(1.21)
*fivehttlpr = = SS*	–23.84	25.60	–0.45	–12.63	93.25
	(–2.86)***	(3.04)***	(–2.15)**	(–1.81)*	(1.90)*
*Age*	–0.42	0.34	0.02	–0.08	2.40
	(–1.92)*	(1.25)	(4.51)***	(–0.45)	(3.23)***
*Male*	6.53	–6.70	0.10	4.48	–10.49
	(0.85)	(–0.89)	(0.57)	(1.05)	(–0.39)
*Constant*	70.47	5.80	1.46	31.14	575.02
	(4.75)***	(0.35)	(4.20)***	(2.65)**	(7.99)***
*R* ^2^	0.12	0.14	0.33	0.28	0.38
Observations	60	60	60	31	31

The table presents OLS estimates of the effects of having the short/short (SS) or short/long (SL) version of the serotonin transporter gene, relative to having the long/long version (LL), on several financial choices and outcomes. Standard errors are adjusted for heteroskedasticity (t-statistics are in parentheses).

### Wealth and Income

Findings in Table 2 suggest that 5-HTTLPR short allele carriers are more conservative in their financial decisions relative to those carrying two copies of the long allele. One possible explanation for these findings is that these groups differ in terms of their wealth, income, or credit constraints. The regression models presented in Table 3 investigate this possibility. As before, we predicted financial variables using only exogenous regressors: the 5-HTTLPR genotype, age, and sex. We did not find any significant differences between the SS, SL or LL subjects in terms of assets, debt, wealth (computed as assets minus debt), income, available credit and used credit (the latter two measures were only available for the subsample of 31 subjects whose credit reports were obtained). As expected, we found that older subjects had significantly more assets and net wealth. Also, males in the sample had higher incomes than females. The lack of an effect of genotype on these wealth-related variables indicates that previously observed differences in financial choices of SS, SL and LL subjects must be driven by other mechanisms.

**Table 3:** The wealth channel.

Dependentvariable	Assets	Debt	Wealth = Assets-Debt	Income	Available	Used
					Credit ($mil)	Credit ($mil)
*fivehttlpr = = SL*	–1.66	–0.65	–1.01	–1.26	–0.05	–0.02
	(–1.27)	(–0.33)	(–0.47)	(–1.24)	(–0.33)	(–0.11)
*fivehttlpr = = SL*	–1.39	–2.58	1.18	0.47	–0.10	–0.13
	(–0.99)	(–1.45)	(0.59)	(0.52)	(–0.54)	(–0.84)
*Age*	0.14	–0.01	0.15	–0.00	–0.00	–0.01
	(3.11)***	(–0.28)	(3.58)***	(–0.11)	(–0.90)	(–1.30)
*Male*	0.69	0.76	–0.07	1.44	0.09	0.06
	(0.67)	(0.55)	(–0.05)	(2.44)**	(0.72)	(0.65)
*Constant*	5.21	9.29	–4.09	6.22	0.55	0.52
	(1.80)*	(3.49)***	(–1.33)	(4.28)***	(1.53)	(1.65)
*R* ^2^	0.36	0.05	0.18	0.13	0.06	0.10
Observations	60	60	60	60	31	31

The dependent variables in the OLS models presented in the table are measures of self-reported assets, debt and net wealth, as well as objective measures of available credit and credit used according to the subjects’ credit reports. Independent variables include the age, sex, as well as the serotonin transporter gene type: SS, SL and LL (omitted category). Standard errors are adjusted for heteroskedasticity (t-statistics are in parentheses).

### Cognitive Abilities and Learning in Financial Markets

It is possible that the 5-HTTLPR short allele carriers are more financially conservative due to varying cognitive skills and abilities to learn in financial markets, since researchers have suggested that serotonin function may also be related to cognitive inflexibility [27]. For example, relative to LL subjects, SS or SL subjects may have lower numeracy scores or may be unable to learn in complex and dynamic environments where optimal choices change over time. These subjects may also have acquired less education and so may have a more limited understanding of financial markets. Such differences could lead short allele carriers to rationally avoid risky or complex financial strategies. Nonetheless, as shown in Table 4, the three groups of subjects did not differ in terms of numeracy, cognitive flexibility, or years of education. The only noticeable difference between groups involved working memory, with SS and SL subjects having slightly lower scores than the LL subjects. While this might present a handicap in certain life domains, we found that it did not correlate with subjects’ abilities to learn from new information in the financial investment task used in this study. Also, short allele carriers did not attend to different types of information in the financial task than did long allele carriers. These results are shown in the last two columns in Table 4. Thus, subjects’ ability to learn from either gain or loss information did not depend on their 5-HTTLPR genotype. The overall learning rate also did not significantly differ across the SS, SL and LL groups. Across the entire sample, subjects made correct (i.e., Bayesian) choices in 62% of trials in the gain condition, and in 66% of the trials in the loss condition (see Table 1).

**Table 4:** The cognitive ability and learning channel.

Dependentvariable	Numeracy	Cognitiveinflexibility	Workingmemory	Years ofeducation	Gainlearning	Losslearning
*fivehttlpr = = SL*	–0.13	8.35	–2.64	1.20	0.24	–0.12
	(–0.17)	(1.22)	(–2.53)**	(1.25)	(1.42)	(–1.54)
*fivehttlpr = = SL*	0.09	–4.40	–2.23	0.40	0.24	–0.08
	(0.12)	(–0.71)	(–2.21)**	(0.48)	(1.45)	(–1.12)
*Age*	–0.01	0.19	–0.05	0.00	0.00	–0.00
	(–0.76)	(2.15)**	(–2.66)**	(0.07)	(1.43)	(–2.28)**
*Male*	0.37	0.44	0.25	0.81	0.03	–0.00
	(0.95)	(0.13)	(0.35)	(1.30)	(0.32)	(–0.02)
*Constant*	8.23	24.91	15.18	14.36	0.16	0.96
	(7.32)***	(3.47)***	(9.64)***	(11.04)***	(0.69)	(7.62)***
*R* ^2^	0.03	0.29	0.23	0.08	0.12	0.18
Observations	60	60	60	60	49	49

The dependent variables in the OLS models presented in the table include numeracy, cognitive inflexibility, working memory, education, and measures of gain and loss learning from the financial investing task. Independent variables include the age, sex, as well as the serotonin transporter gene type: SS, SL and LL (omitted category). Standard errors are adjusted for heteroskedasticity (t-statistics are in parentheses).

### Beliefs About Risk and Self

Another possibility is that genotype influences subjects’ beliefs about the risk of financial investing or their own ability to invest. Findings provided some support for this interpretation. As the results in Table 5 show, controlling for age and sex, SS subjects were more likely to perceive stocks as very risky, relative to LL subjects (although at weak significance levels). At the same time, SS subjects were significantly more likely to report having lower socio-economic status, either in their local community (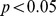) or relative to the U.S. population as a whole (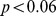). Since there were no significant differences between SS subjects and the other groups in terms of wealth, income, or education (including sex and age as controls), these findings suggest that short allele carriers were subjectively more pessimistic when assessing their standing in society (their status, ability or success). To the extent that low socio-economic status perceptions correlate with, or induce, pessimistic beliefs regarding one’s abilities across other domains, this result suggests that 5-HTTLPR short allele carriers may be more likely to doubt their ability as investors, compared to other individuals. Also, prior findings that low socio-economic status correlates with less perceived control [17] may provide another explanation as to why long allele carriers (who score higher in perceived socio-economic status) may be more willing to invest in risky assets. The control variables used in the regressions in Table 5 confirmed this line of reasoning. Relative to female subjects, males perceived stocks to be less risky. Older adults believed that they ranked higher in terms of socio-economic status, consistent with the finding in Table 3 that they possess higher wealth relative to younger subjects.

**Table 5:** The beliefs channel.

Dependentvariable	Perception ofrisk of stocks	Perception of one’s statusin the local community	Perception of one’s statusin the US
*fivehttlpr = = SL*	0.67	–0.43	–0.87
	(1.17)	(–0.77)	(–1.99)**
*fivehttlpr = = SL*	0.83	–1.04	–0.69
	(1.67)	(–2.28)**	(–1.91)*
*Age*	0.01	0.02	0.03
	(0.50)	(1.75)*	(3.13)***
*Male*	–0.94	0.40	0.42
	(–2.48)**	(1.08)	(1.30)
*Constant*	4.75	5.81	5.39
	(4.86)***	(8.25)***	(10.61)***
*R* ^2^	0.16	0.18	0.20
Observations	60	60	60

The dependent variables in the OLS models presented in the table are the subjects’ beliefs regarding the risk of investing in stocks, and their perception of status in their local community and in the US as a whole. Independent variables include the age, sex, as well as the serotonin transporter gene type: SS, SL and LL (omitted category). Standard errors are adjusted for heteroskedasticity (t-statistics are in parentheses).

### Neuroticism, Positive Affect, and Negative Affect

The link between the 5-HTTLPR gene variants and financial choices may also be related to affect, since prior work suggests that manipulations of the serotonin system in the brain can influence people’s emotional state [28]. In light of the evidence in prior section, it is possible that short allele carriers, relative to long allele carriers, are more anxious about facing risky outcomes, and as a result, chose to avoid risky financial strategies. Findings strongly supported this mechanism in several ways.

First, allelic variation correlates robustly with individual differences in subjects’ neuroticism, as shown in the regressions in Table 6. SS and SL subjects had were significantly more neurotic than LL subjects (6 points difference, 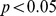), in line with results from prior genetic association studies (e.g., [11]). This represents a large effect, given that the mean and standard deviation of neuroticism in the sample were 27.93 and 8.98, respectively (see Table 1). Subjects in the SS and SL groups also reported experiencing more negative affect than subjects in the LL group (0.2 points difference, 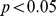), and less positive affect (0.4 points difference for the SS group, 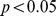). These effect sizes were comparable to one standard deviation for each of the measures (i.e., 0.40 points for negative affect and 0.52 points for positive affect, see Table 1).

**Table 6:** Genetic effects on anxiety and affect.

Dependent variable	Neuroticism	Negative Affect	Positive Affect
*fivehttlpr = = SL*	5.50	0.26	–0.32
	(2.26)**	(2.36)**	(–1.67)
*fivehttlpr = = SL*	5.87	0.21	–0.42
	(2.63)**	(2.23)**	(–2.49)**
*Age*	–0.04	0.00	0.01
	(–0.59)	(0.35)	(1.74)*
*Male*	–6.37	–0.15	−0.05
	(–2.84)***	(–1.49)	(−0.42)
*Constant*	28.44	1.70	2.97
	(6.70)***	(8.15)***	(11.14)***
*R* ^2^	0.22	0.08	0.16
Observations	60	60	60

The dependent variables in the OLS models presented in the table are the subjects’ neuroticism, negative and positive affect scores. Independent variables include the age, sex, as well as the serotonin transporter gene type: SS, SL and LL (omitted category). Standard errors are adjusted for heteroskedasticity (t-statistics are in parentheses).

Second, financial choices were specifically related to the component of the neuroticism score attributable to 5-HTTLPR genotype. In the first column of Table 7 the neuroticism score was regressed onto dummy variables indicating whether a subject’s genotype was SS, SL or LL. This regression yielded two orthogonal components of neuroticism: that driven by the 5-HTTLPR genotype, and a residual component (i.e., predicted values and residuals in the regression in the first column). Regression models similar to those in Table 2 were estimated to determine whether the component of neuroticism driven by the 5-HTTLPR genotype, as well as the residual neuroticism, correlated with subjects’ allocations to stocks, cash, their involvement in financial decisions, their number of credit lines, and their FICO score. Results indicated that the component of neuroticism specifically driven by subjects’ 5-HTTLPR allelic variation significantly and robustly predicted all five measures of financial choices and outcomes. The residual component of neuroticism, however, only correlated with one of the five measures – involvement in finances. The sign of all these effects was consistent with the predicted anxiety mechanism, such that subjects with higher values of the genetically-driven component of the anxiety measure invested a lower fraction of money in stocks, kept a higher fraction in cash, were less actively involved in complex financial decisions, had fewer credit lines, and had higher credit scores.

**Table 7:** Genetically-driven effects of anxiety on financial choices and outcomes.

Dependentvariable	Neuroticism	%StockAllocation	%CashAllocation	Involvementin Finances	Number ofcredit lines	FICOscore
*fivehttlpr = = SL*	4.18					
	(1.71)*					
*fivehttlpr = = SL*	7.40					
	(3.01)***					
*Neuroticism^Predicted^*		–3.46	3.76	–0.06	–1.88	12.90
		(–2.96)***	(3.03)***	(–2.09)**	(–1.82)*	(1.92)*
*Neuroticism^Residual^*		–0.59	0.24	–0.03	–0.05	0.95
		(–1.27)	(0.46)	(–2.13)**	(–0.14)	(0.80)
*Age*		–0.38	0.22	0.02	–0.18	2.26
		(–1.81)*	(0.91)	(3.75)***	(–1.14)	(3.39)***
*Male*		3.40	–6.53	–0.11	2.96	–4.02
		(0.44)	(–0.83)	(–0.70)	(0.57)	(–0.15)
*Constant*	22.67	150.96	–77.83	3.04	78.78	285.08
	(13.34)***	(4.04)***	(–1.97)**	(3.50)***	(2.31)**	(1.30)
*R* ^2^	0.09	0.14	0.12	0.36	0.24	0.39
Observations	60	60	60	60	31	31

The first column in the table presents the results of an OLS regression of the neuroticism score on dummy variables indicating the subjects’ serotonin transporter gene variant (SS, SL, LL, the latter being the omitted category). The predicted and residual values of neuroticism obtained from this regression, as well as age and sex, are used as explanatory variables in the OLS models in the next five columns, in which the dependent variables are measures of the subjects’ financial choices and outcomes (same as in Table 2). Standard errors are adjusted for heteroskedasticity (t-statistics are in parentheses).

To verify the robustness of these effects, in Table 8 we estimated similar regression models as in Table 7 but included the subjects’ wealth, cognitive ability (as measured by the performance on the Trail Making Test of cognitive flexibility) and beliefs regarding the riskiness of investing in stocks as additional control variables. Even with the inclusion of these controls, the effects of the genetically-driven neuroticism on avoiding risky and complex financial choices were in line with those estimated in the main analysis in Table 7. The control variables themselves had expected effects. For example, subjects with more wealth were more actively involved in making financial decisions and had higher FICO scores, while those who had stronger beliefs that stocks were risky investments in fact allocated a smaller fraction of their money into stocks, and a larger fraction into cash. Using different proxies for cognitive ability and beliefs yielded results similar to those documented in Table 8.

**Table 8:** The role of additional controls on the genetically-driven effects of anxiety on financial choices and outcomes.

Dependentvariable	%StockAllocation	%CashAllocation	Involvement in Finances	Numberof creditlines	FICOscore
*Neuroticism^Predicted^*	–3.29	3.65	–0.06	–1.43	10.43
	(–3.49)***	(3.50)***	(–2.14)**	(–1.77)*	(1.63)
*Neuroticism^Residual^*	–0.62	0.17	–0.02	–0.01	0.71
	(–1.52)	(0.36)	(–1.83)*	(–0.04)	(0.62)
*Age*	–0.31	0.34	0.01	–0.26	1.57
	(–1.21)	(1.33)	(2.13)**	(–1.41)	(1.81)*
*Male*	–2.08	–1.63	–0.12	–1.86	–10.62
	(–0.27)	(–0.19)	(–0.72)	(–0.41)	(–0.39)
*Wealth*	1.05	–2.17	0.04	–0.13	5.05
	(1.25)	(–3.02)***	(2.65)**	(–0.37)	(2.33)**
*Cognitive* *inflexibility*	–0.45	0.29	0.00	0.22	–0.19
	(–1.98)*	(1.17)	(0.41)	(1.21)	(–0.18)
*Belief about* *risk*	–6.91	7.00	–0.03	–3.46	–7.48
*of stocks*	(–3.55)***	(3.12)***	(–0.64)	(–2.62)**	(–0.83)
*Constant*	193.59	–122.21	3.29	84.49	409.79
	(6.61)***	(–3.37)***	(3.72)***	(2.97)***	(2.02)**
*R* ^2^	0.28	0.32	0.42	0.44	0.49
Observations	60	60	60	31	31

The predicted and residual values of neuroticism obtained from the regression in column 1 of Table 7, as well as age, sex, wealth, cognitive inflexibility and beliefs about the risk of investing in stocks are used as explanatory variables in the OLS models in each of the five columns in the table, in which the dependent variables are measures of the subjects’ financial choices and outcomes (same as in Table 2). Standard errors are adjusted for heteroskedasticity (t-statistics are in parentheses).

## Discussion

**Figure 1:**
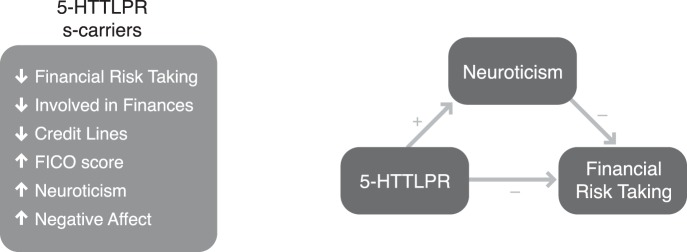
5-HTTLPR genotype, neuroticism, and financial choices. The presence of the short allele of the 5-HTTLPR gene relates to the avoidance of risky and complex financial choices. These genetic effects are mediated by neuroticism.

In a community sample of healthy adults, we found that individuals who carry the 5-HTTLPR short allele were more likely to avoid risk and complexity in real life financial choices. Specifically, short allele carriers invested less in equities, were less actively engaged in making investment decisions, and had fewer credit lines than long allele carriers. These differences were not simply due to differences in wealth, since wealth, assets, debt and income did not differ between groups, and credit (i.e., FICO) scores of short allele carriers were actually higher. Differences in financial decisions between the short and the long allele carriers were also not driven by differences in cognitive skills (e.g., memory, cognitive flexibility or numeracy), education, or the ability to learn from financial information. The differences between short and long allele carriers instead appeared to result from individual differences in emotional reactivity, since short allele carriers have increased neuroticism and negative affect, and decreased positive affect. These findings, summarized in Figure 1, suggest that short allele carriers focus on the negative potential outcomes of their financial choices, and as a result, choose to avoid complex and risky financial investments.

Overall, these results indicate that individual variation in the 5-HTTLPR genotype influences financial choice by modifying attitudes towards financial risk taking by increasing anxiety during anticipation of potential losses, rather than through cognitive ability or learning. The paper contributes to a growing literature concerning the role of physiological factors in financial decision making. A number of large studies now suggest that people’s genetic endowment correlates with investment behavior [2], [3], [4]. Specific heritable traits such as intelligence appear to promote participation in the stock market [29]. Trading performance also correlates with psychophysiological measures of emotional reactivity [30], and levels of circulating hormones like testosterone have been linked to trading behavior and preferences for risky financial jobs [31], [32], [33], [34], [35]. Indeed, the present investigation was specifically inspired by previous laboratory research suggesting that individuals with the short form of the 5-HTTLPR allele are more likely to avoid financial risks [6], [36]. Investigators have also explored the influence of alleles related to the function of other neurotransmitters such as dopamine on financial risk taking, albeit with more mixed results [37], [5], [36], [38]. The evidence documented here not only follows this emerging line of inquiry, but further suggests specific psychological mechanisms by which genetic variation can influence financial choices.

It is important to note that environmental factors (such as personal background and experiences) also play important roles in financial decision making. Economic conditions early in life impact adult risk taking in both individual investors and corporate executives [39], [40]. Financial decision making is also influenced by age [41], [42], [43] and sex [44]. Gene-environment interactions are also likely to have significant influence. For example, the finding that individuals who have the 5-HTTLPR short allele are more likely than others to develop depression in response to adverse life events [45] suggests that people with different gene variants may cope differently with negative economic shocks. Future studies of physiological (neural, genetic or hormonal) influences on economic choice thus may strive to pinpoint intervening mechanisms responsible for links between physiological factors and economic behavior. This work aimed to contribute to this new research direction by illuminating one set of mechanisms that connects genetics to financial decisions.

While our findings indicate that genetic influences on financial choice act at least in part by altering investors’ affective reactions towards risk, certain limitations of the research must be acknowledged. First, some outcome variables utilized self-reported and survey responses to hypothetical investment questions. We partially addressed this concern by verifying that the self-reported financial measures concerning personal debt and assets correlated well with actual objective values obtained from the subjects’ credit reports. Nonetheless, the findings may still be influenced by a selection bias, since subjects who provided access to their credit reports may also have provided the most accurate self-reported financial measures. With respect to the reliability of survey data concerning financial decisions, such data is commonly used in the empirical finance literature in order to explore managerial and household decisions [46], [47]. Second, these findings come from a restricted sample of individuals. Researchers have raised concerns about the influence of sample size on limiting power in studies of genetic associations [48], but have also noted the potential favorable impact of linking to intermediate phenotypic processes, which could improve power without inflating sample sizes [49]. Traditionally, studies have attempted to link genetic variance to psychiatric diagnoses, which suffer from limited reliability and validity. As crystallized measures of repeated financial decisions, real life financial outcomes may exhibit greater reliability and validity than psychiatric diagnoses and thus confer greater power for testing genetic linkages, especially when clear ex-ante hypotheses can be formulated based on prior results, as in this study. While presently the cost of genotyping and data collection may limit the number of subjects in a study as ours, future studies may continue to explore these associations in larger and more general samples. Regardless, the effect of the 5-HTTLPR genotype on portfolio allocation decisions is consistent with previous findings [6], which were obtained in a different experiment, using a different subject pool and provider of genotyping services, implying generalizability. These findings critically extend previous research from the laboratory to real world finance, and isolate a psychological causal mechanism related to chronic negative emotions.

### Conclusion

The primary goal of this research was to elucidate a mechanism by which genetic factors might influence financial choice. Thus, we focused on one genetic variant that laboratory studies have related to financial risk taking – a specific polymorphism in the the promoter region of the serotonin transporter gene (5-HTTLPR). We found that individuals who have the 5-HTTLPR short allele avoid risky and complex financial decisions, even though they do not differ from others in terms of wealth, income, assets, debt, education, cognitive abilities, numeracy, or learning based on financial information. Instead, a mechanism responsible for the effects of the short allele on financial choice appears to involve people’s negative affective reactions when anticipating upcoming investment decisions. Specifically, relative to other subjects, short allele carriers perceived stocks to be riskier and were more anxious and prone to feeling negative emotions, suggesting that these individuals tend to experience higher anxiety when faced with risky investments involving potential losses.

These findings thus make several novel contributions. First, we identified a mechanism through which a specific genetic factor (5-HTTLPR) influences financial choice, rather than merely documenting correlations between genetic variants and behavior. Second, we acquired integrative genetic, survey, and experimental data in a community sample of adults, and corroborated this data with objective information from individuals’ credit reports. Together, these findings suggest that studying the biological aspects of decision making can help explain individuals’ immediate economic choices and eventual life financial outcomes.
